# Clinical findings in relation to mortality in non-tuberculous mycobacterial infections: patients with *Mycobacterium avium* complex have better survival than patients with other mycobacteria

**DOI:** 10.1007/s10096-015-2432-8

**Published:** 2015-07-09

**Authors:** H. Kotilainen, V. Valtonen, P. Tukiainen, T. Poussa, J. Eskola, A. Järvinen

**Affiliations:** University of Helsinki and Division of Infectious Diseases, Inflammation Center, Department of Medicine, Helsinki University Hospital, Helsinki, Finland; University of Helsinki and Division of Lung Diseases, Department of Medicine, Helsinki University Central Hospital, Helsinki, Finland; STAT-Consulting, Nokia, Finland; University of Helsinki and Mycobacteriology Unit, Helsinki University Hospital Laboratory (HUSLAB), Helsinki, Finland

## Abstract

We compared the clinical findings and survival in patients with *Mycobacterium avium* complex (MAC) and other non-tuberculous mycobacteria (NTM). A total of 167 adult non-human immunodeficiency virus (HIV) patients with at least one positive culture for NTM were included. Medical records were reviewed. The patients were categorised according to the 2007 American Thoracic Society (ATS) criteria. MAC comprised 59 % of all NTM findings. MAC patients were more often female (70 % vs. 34 %, *p* < 0.001) and had less fatal underlying diseases (23 % vs. 47 %, *p* = 0.001) as compared to other NTM patients. Symptoms compatible with NTM infection had lasted for less than a year in 34 % of MAC patients but in 54 % of other NTM patients (*p* = 0.037). Pulmonary MAC patients had a significantly lower risk of death compared to pulmonary other NTM (hazard ratio [HR] 0.50, 95 % confidence interval [CI] 0.33–0.77, *p* = 0.002) or subgroup of other slowly growing NTM (HR 0.55, 95 % CI 0.31–0.99, *p* = 0.048) or as rapidly growing NTM (HR 0.47, 95 % CI 0.25–0.87, *p* = 0.02). The median survival time was 13.0 years (95 % CI 5.9–20.1) for pulmonary MAC but 4.6 years (95 % CI 3.4–5.9) for pulmonary other NTM. Serious underlying diseases (HR 3.21, 95 % CI 2.05–5.01, *p* < 0.001) and age (HR 1.07, 95 % CI 1.04–1.09, *p* < 0.001) were the significant predictors of mortality and female sex was a predictor of survival (HR 0.38, 95 % CI 0.24–0.59, *p* < 0.001) in the multivariate analysis. Pulmonary MAC patients had better prognosis than pulmonary other NTM patients. The symptom onset suggests a fairly rapid disease course.

## Introduction

The incidence of non-tuberculous mycobacteria (NTM) in clinical samples has been reported to increase, and they have become a more common finding than classical *Mycobacterium tuberculosis* in many countries [1–3]. *M. avium* complex (MAC) has been the most commonly found NTM in human infections in most parts of the world, but in Europe, it makes up only about one-third of NTM isolations [3–6]. Other NTM species in clinical findings are all clearly less common but also variable in different geographical regions. In Europe, *M. gordonae* consisted of up to 17 %, rapidly growing mycobacteria (RGM) 16 %, *M. xenopi* 14 % and *M. malmoense* 1 % of all NTM isolations in 2008 [6]. However, in northern Europe, *M. malmoense* has been the second or third most common isolate [7–9]. In other parts of the world, *M. kansasii*, *M. abscessus* and *M. chelonae* have been common isolations and, especially in Asia, RGM have been commonly reported [4–6]. During the last year, the interest in NTM infections has increased, with the result of almost an explosion of published articles. NTM have been reported more often in patients with underlying diseases like autoimmune diseases and bronchiectasis or cystic fibrosis [1, 10–13]. Almost 10 % of patients with bronchiectasis have been observed to have NTM and NTM have been suggested to increase the risk of chronic obstructive lung disease [12, 14]. Furthermore, NTM have been reported to be associated with an increased proportion of deaths in USA and increased risk of respiratory failure [15, 16].

MAC has generally been considered to be more pathogenic and more often disease-causing than the other NTM strains [17–19]. This has resulted in the American Thoracic Society (ATS) publishing criteria to discern NTM pulmonary disease from colonisation and the criteria were designed for MAC, *M. kansasii* and *M. abscessus* [17]. However, recent studies have shown that ATS criteria did not predict fatal outcome in patients with an NTM isolation [1, 10]. In a population-based study from Denmark, *M. xenopi* was associated to poorer prognosis than MAC [1]. Furthermore, ATS criteria-positive MAC patients had total mortality of 24 % but MAC-specific mortality was as low as 5 % [20]. A very recent study observed that a single isolation of *M. fortuitum* in a respiratory specimen seldom indicated progression of disease and, after single isolations of other NTM species, progression was dependent on patient age and radiographic changes, but not on the NTM species [21]. Indeed, the prognostic value of non-MAC NTM isolation still seems to be largely unclarified.

The clinical picture of disease caused by various NTM species has been divergent and even stereotypic in the literature. MAC has been reported to affect more often female non-smokers without underlying diseases and to cause either pulmonary nodules or bronchiectasis with less cavities [7, 20, 22]. Patients with *M. xenopi* infections have generally been described as middle-aged smoking males with chronic obstructive pulmonary disease (COPD) or prior tuberculosis with upper lobe cavitary infections [23]. *M. malmoense* has been linked to fibrocavitary disease with tuberculosis or MAC-like symptoms in males with COPD [6]. *M. kansasii* has been linked to disease indistinguishable from tuberculosis, with a clear male predominance [24, 25]. However, several recent follow-up studies have indicated that pulmonary NTM findings carry a high mortality rate, ranging from 29 to 69 % within 5 years [1, 7, 20]. Most data from disease due to various NTM species come from studies collecting data from patients with one particular NTM. Prognosis, symptoms and underlying factors as compared to MAC with similar setting and follow-up are scarce.

The aim of this study was to ascertain underlying diseases, symptoms and prognostic factors in relation to mortality in NTM infections. In this study, firstly, we retrospectively followed patients with an NTM isolation for, on average, 7 years. Secondly, we evaluated the survival, clinical differences and characteristics in NTM infections between MAC and other NTM.

## Materials and methods

### Study population

Human immunodeficiency virus (HIV)-negative patients with at least one positive culture finding for NTM from 1990 to the end of 1998 and from 2004 to the end of 2009 were included in the study. Samples for mycobacterial culture were analysed by the Central Microbiological Laboratory of Helsinki City, later Helsinki University Central Hospital Laboratory (HUSLAB), and patients were identified and matched with their records and isolates by using their unique personal identity number; these numbers are given to all residents of Finland.

All patients had been referred to a pulmonary specialist in some of the eight different hospitals in the area of Helsinki University Central Hospital because of pulmonary symptoms compatible with NTM infection. One patient with concomitant findings of *M. tuberculosis* and *M. avium* was excluded. Altogether, 167 patients (adults aged 16–94 years) were included in the study population. The patient material has been, in part, presented in our previous studies [7, 26]. For analyses of pulmonary NTM infection, all six patients with a cutaneous NTM isolation were excluded. The numbers of subsequent positive or negative mycobacterial smears, cultures and sampling sites were recorded. Demographic characteristics (age, sex, occupation, body weight, height) were obtained from the patient records. The concomitant diseases were classified as described by McCabe and Jackson [27] as follows: (1) healthy, i.e. no other diseases; (2) chronic non-fatal diseases; (3) ultimately fatal diseases with expected life expectancy of 5 years maximum, such as carcinoma with local spreading and uncompensated hepatic cirrhosis; (4) rapidly fatal diseases with expected survival of no more than 6 months, such as carcinoma with widespread metastases. In particular, information on previous pulmonary diseases, such as bronchiectasis, COPD, pulmonary fibrosis, prior tuberculosis, asthma, pulmonary malignancies and pneumonias, were retrieved. The signs, symptoms, microbiological data and other laboratory findings were all reviewed at the time of the first positive NTM culture and at patient visits closest (6 months) to 1 year after the positive culture. The radiological findings of chest X-ray, computed tomography (CT) scans and high-resolution computed tomography (HRCT) were collected from the original radiologist statements at patient visits closest (6 months) to 1 year after the positive culture and classified as infiltrates, nodules, cavities or bronchiectasis according to the 2007 ATS criteria [17]. Only data relevant to ATS criteria were collected, and in unclear cases, a radiologist was consulted. Previous (6 months) immunosuppressive treatments were reviewed. Systemic or inhalation corticosteroid treatment was recorded when continued for longer than 1 month. Information on previous pulmonary diseases was retrieved. Data on smoking habits were retrieved from the medical records and pulmonary function test questionnaires. The date of death was ascertained from the Finnish Population Register Centre records or patient records.

### Microbiological methods

Clinical specimens, except for blood samples, were stained with auramine-O-fluorochrome dye and examined microscopically for acid-fast bacilli (AFB). Cultures positive for AFB were identified by conventional biochemical tests and hybridisation with DNA probes (AccuProbe; Gen-Probe, San Diego, CA, USA). Cultures negative for *M. tuberculosis* and MAC were identified by amplification and sequencing of the 16S rRNA gene. For those NTM patients with a positive culture finding for NTM from 2004 to the end of 2009, cultures positive for AFB were identified by DNA strip assays (GenoType Mycobacterium CM/AS, Hain Lifescience, Nehren, Germany). MAC, *M. avium*, and *M. intracellulare* were all classified as MAC due to changing nomenclature during the study period.

### Case definitions

The NTM patients were categorised according to the 2007 ATS criteria [25] in order to reveal how many fulfilled the ATS diagnosis for NTM disease. ATS-positive patients had to meet microbiological, radiological and symptoms criteria. According to these diagnostic criteria, a patient should have the NTM isolated in at least two sputum cultures or one positive culture from a bronchoscopy sample (by lavage or by brush) or from a lung biopsy to fulfill the microbiological criteria. One positive culture from a skin or lymphatic tissue biopsy fulfilled the microbiological criteria for extrapulmonary disease. Radiographic criteria were fulfilled when nodular or cavitary opacities were found on chest radiographs or CT, or when bronchiectasis with multiple small nodules were found by CT/HRCT. The patient had to have symptoms compatible with NTM disease and they included: cough, dyspnoea, fatigue, fever, weight loss, haemoptysis or decreased appetite. If the patient did not meet these microbiological, radiographic and symptomatic criteria, he/she was categorised as ATS-negative.

### Statistical methods

The patients having MAC were compared to patients with other NTM strains. The independent samples *t*-test was used for continuous variables. The Mann–Whitney *U*-test was used if the assumption of normality was not achieved using transformations. The Chi-squared test and Fisher’s exact test were used for categorical variables. The Kaplan–Meier method was used to estimate the median survival times with 95 % confidence intervals (CIs). The univariate Cox proportional hazards model was used to compare the survival times of the MAC patients to the patients with other NTM strains. Also, the survival times between different mycobacterial species and groups of species were compared. The results are given as hazard ratios (HRs) with 95 % CIs. In addition, two predefined adjusted survival time comparisons between MAC and other NTM strains were conducted, including ATS 2007 positivity (positive vs. negative) and underlying diseases according to the McCabe classification (3–4 vs. 1–2) as additional dichotomous covariates. The interactions were tested including appropriate interaction terms in the models. In the next phase, smoking, fulfilment of ATS 2007 criteria, underlying diseases according to the McCabe classification (1–2 or 3–4), age, gender and smoking were considered as potential additional predictors, as they were all significant predictors in the univariate models. They were introduced to the forward stepwise (criterion for entry *p* < 0.05) multivariate Cox proportional hazards model. The assumption of proportional hazards was graphically assessed. We obtained plots of log (log S(t)) versus time in order to see the difference between the survival curves. If the survival curves were approximately parallel, then the proportionality assumption was reasonable. Based on those graphical plots, the proportionality assumption was not rejected for any predictor. The number of multivariate analyses was three. They were performed according to the predefined analysis plan. Other multivariate analyses were mainly exploratory or stepwise Cox models using different variable selection methods (different cut-off values for *p*-values and different blocks of variables). These additional models were performed in order to confirm the results. The predictors in the three final models were: (1) multivariate model with two predictors: fulfilment of MAC and fulfilment of ATS 2007 criteria (Fig. 2, panel A). (2) Multivariate model with two predictors: fulfilment of MAC and underlying diseases according to the McCabe classification (Fig. 2, panel B). (3) Age, gender, smoking, McCabe classification, ATS 2007 fulfilment and MAC fulfilment were introduced as potential predictors to the stepwise multivariate model. Three predictors were selected: McCabe classification, age and gender. Analysis were performed using IBM SPSS Statistics for Windows (version 21.0; IBM Corp., Armonk, NY, USA).

## Results

### Mycobacterial strains and patient characteristics

MAC comprised the majority (99/167, 59 %) of all NTM findings. Other NTM were isolated in 68 (41 %) patients. RGM *M. fortuitum*, *M. chelonae* and *M. abscessus* comprised 13 % (22/167) of all NTM findings and *M. malmoense* 9 % (15/167). *M. gordonae* was found in 10 % (16/167), *M. xenopi* in 2 % (4/167), *M. marinum* and *M. terrae* in 1 % of cases each (2/167). *M. paraffinicum* and *M. triplex* were both cultured in one patient (1 %) and other non-specified NTM species in five patients (3 %). Two cases of both *M. fortuitum* and *M. marinum* were cultured from skin, like one *M. chelonae* and one non-specified NTM species, which were not included in the mortality analyses for pulmonary NTM infection.

The mean age of the patients was 66 (range 16–94) years, with no difference between MAC and other NTM patients (Table 1). The majority of MAC patients were female (70 %), whereas only 34 % of patients with other NTM were female (*p* < 0.001). MAC patients were more lean than other NTM patients, as assessed by the body mass index (BMI, 20.3 kg/m^2^ vs. 22.9 kg/m^2^, respectively, *p* = 0.001). There were also other significant differences between MAC and other NTM patients; MAC patients had less often fatal underlying diseases (23 % vs. 47 %) or a malignancy (12 % vs. 22 %) but more often bronchiectasis (25 % vs. 7 %), more often inhalation corticosteroid use (27 % vs. 13 %) and they were more often non-smokers (58 % vs. 29 %) as compared to patients with other NTM (Table 1).

**Table 1:** Characteristics and underlying diseases of 167 patients with at least one isolation of non-tuberculous mycobacteria (NTM) categorised according to *Mycobacterium avium* complex (MAC) and other NTM species

	MAC, *n* = 99		Other NTM, *n* = 68		Total, *n* = 167		*p*-Value
	*n* ^b^	(%)	*n* ^b^	(%)	*n* ^b^	(%)	
Age, mean years (SD)	65.6	(14.4)	66.7	(13.3)	66.0	(13.9)	0.62^a^
BMI^c^, kg/m^2^ mean (SD)	20.3	(3.7)	22.9	(4.3)	21.3	(4.2)	0.001^a^
Female	69	(70)	23	(34)	92	(55)	<0.001^d^
Underlying diseases^e^							
Healthy or non-fatal disease	76	(77)	36	(53)	112	(67)	0.001^d^
Ultimately or rapidly fatal disease	23	(23)	32	(47)	55	(33)	
Underlying pulmonary diseases							
Bronchiectasis	25	(25)	5	(7)	30	(18)	0.003^d^
COPD^f^	23	(23)	21	(31)	44	(26)	0.27^d^
Prior tuberculosis	16	(16)	13	(19)	29	(17)	0.62^d^
Lung fibrosis	8	(8)	9	(13)	17	(10)	0.28^d^
Asthma	14	(14)	11	(16)	25	(15)	0.72^d^
Other pulmonary disease	16	(16)	19	(28)	35	(21)	0.07^d^
Pulmonary or other malignancy	12	(12)	15	(22)	27	(16)	0.09^d^
Any previous pulmonary diseases	72	(73)	52	(76)	124	(74)	0.59^d^
Autoimmune diseases	11	(11)	10	(15)	21	(13)	0.49^d^
No alcohol abuse	94	(95)	60	(88)	154	(92)	0.11^d^
Non-smoker	57	(58)	20	(29)	77	(46)	<0.001^d^
Corticosteroid >1 month	25	(26)	18	(27)	43	(26)	0.85^d^
Corticosteroid inhalation >1 month	26	(27)	9	(13)	35	(21)	0.04^d^
Immunosuppressive therapy	8	(8)	8	(12)	16	(10)	0.43^d^
ATS 2007-positive	60	(61)	33	(49)	93	(56)	0.12^d^

^a^ *t*-Test for independent samples

^b^Values are expressed as *n* (%), unless otherwise stated

^c^Body mass index, kg/m^2^

^d^Chi-squared test

^e^Underlying diseases classified according to the criteria of the McCabe classification [27]: (1) healthy, i.e. no other diseases; (2) non-fatal chronic diseases; (3) ultimately fatal diseases with maximum life expectancy of 5 years; (4) rapidly fatal diseases with expected survival for no more than 6 months

^f^Chronic obstructive pulmonary diseases

### Symptoms and clinical findings on presentation

Symptoms compatible with NTM disease had lasted for less than a year in 34 % of MAC patients but in 54 % of patients with other NTM (*p* = 0.01, Table 2). Cough was the most common symptom, reported by 77 % of all patients. MAC patients had more often systemic symptoms like fever (48 % vs. 31 %, *p* < 0.02) and fatigue (47 % vs. 32 %, *p* = 0.05) as compared to patients with other NTM.

**Table 2:** Symptoms and signs at the time of the first positive NTM isolation in 167 patients categorised according to MAC and other NTM species

	MAC, *n* = 99		Other NTM, *n* = 68		Total, *n* = 167		*p*-Value
	*n* ^b^	(%)	*n* ^b^	(%)	*n* ^b^	(%)	
Duration of symptoms							
<1 year	33	(34)	37	(54)	70	(43)	0.01^a,c^
1–2 years	35	(36)	18	(26)	53	(32)	
3–10 years	17	(18)	10	(15)	27	(16)	
>10 years	11	(12)	3	(4)	14	(9)	
Respiratory symptoms							
Cough	79	(80)	49	(72)	128	(77)	0.25^a^
Dyspnoea	53	(54)	32	(47)	85	(51)	0.41^a^
Haemoptysis	25	(25)	13	(19)	38	(23)	0.35^a^
Systemic symptoms							
Fatigue	47	(47)	22	(32)	69	(41)	0.05^a^
Fever >37.5 °C	48	(48)	21	(31)	69	(41)	0.02^a^
Weight loss	32	(32)	17	(25)	49	(29)	0.31^a^
Non-specific symptoms							
Decreased appetite	13	(13)	11	(16)	24	(14)	0.58^a^
Palpitation	12	(12)	9	(13)	21	(13)	0.83^a^
Chest pain	14	(14)	10	(15)	24	(14)	0.92^a^
Arthralgia	7	(7)	5	(8)	12	(7)	1.00^d^
Night sweats	7	(7)	1	(1)	8	(5)	0.14^d^
Lymphadenitis	4	(4)	4	(6)	8	(5)	0.72 ^d^

^a^Chi-squared test

^b^Values are expressed as *n* (%) patients with valid information, unless otherwise stated

^c^The categories 1–2, 3–10 and >10 years were combined before analysis

^d^Fisher’s exact test

Only one patient with *M. marinum* isolated from skin had normal chest X-ray, whereas all the other patients in both groups who had chest X-ray taken had pathological findings (Table 3). There were significantly more patients with nodules in CT or HRCT in the MAC group (34 %) compared with the other NTM group (13 %, *p* = 0.003, Table 3). Also, bronchiectasis was more common in MAC than other NTM patients (31 % vs. 13 %, *p* = 0.009, Table 3), whereas infiltrates (42 % vs. 40 %) and cavities (10 % vs. 6 %) were not significantly different between the groups. The radiological findings were concentrated in the right upper and middle lobes in both groups, without any significant difference (Table 3). Diffuse pulmonary findings in the MAC group were more common than in the other NTM group (29 % vs. 15 %, *p* = 0.03, Table 3).

**Table 3:** Radiological findings during the first year after NTM isolation in 167 patients categorised according to MAC and other NTM species

	MAC, *n* = 99		Other NTM, *n* = 68		Total, *n* = 167		*p*-Value
	*n* ^b^	(%)	*n* ^b^	(%)	*n* ^b^	(%)	
Radiological examinations							
Normal X-ray	0	(0)	1	(1.5)	1	(1)	0.40^e^
Abnormal X-ray	93	(94)	61	(90)	154	(92)	
No X-ray	6	(6)	6	(9)	12	(7)	0,.55^e^
No CT or HRCT^c^	44	(44)	32	(47)	76	(46)	0.74^a^
Abnormal CT or HRCT	55	(56)	36	(53)	91	(55)	
Radiographical findings^d^							
Infiltrates	39	(42)	25	(40)	64	(41)	0.84^a^
Nodules	32	(34)	8	(12)	40	(26)	0.003^a^
Cavities	9	(10)	4	(6)	13	(8)	0.48^a^
Bronchiectasis	29	(31)	8	(13)	37	(24)	0.009^a^
Missing information	6	(6)	6	(9)	12	(7)	0.55^e^
Location of radiographical findings^d^							
Right upper lobe	36	(38)	21	(31)	57	(35)	0.42^a^
Right middle lobe	27	(28)	17	(25)	44	(27)	0.70^a^
Right lower lobe	17	(18)	10	(15)	27	(17)	0.64^a^
Left upper lobe	13	(14)	13	(19)	26	(16)	0.32^a^
Left lower lobe	20	(21)	15	(22)	35	(21)	0.81^a^
Diffuse findings in both lungs	28	(29)	10	(15)	38	(23)	0.03^a^
Missing information	3	(3)	1	(2)	4	(2)	0.65^e^

^a^Chi-squared test

^b^Values are expressed as *n* (%) patients with valid information

^c^ *CT* computed tomography; *HRCT* high-resolution computed tomography of the lungs

^d^Multiple locations were possible

^e^Fisher’s exact test

There were no differences in elementary laboratory tests like haemoglobin, alkaline phosphatase and alanine transaminase levels between the groups (data not shown). The median C-reactive protein (CRP) was 11.0 mg/L (interquartile range, IQR 2.5–45.0) and the median erythrocyte sedimentation rate was 25 mm/h (IQR 11–58) in the MAC group and the corresponding median CRP and erythrocyte sedimentation rate were 18.0 mg/L (IQR 5.0–42.0) and 21 mm/h (IQR 11–42) in the group with other NTM.

### Mortality and follow-up

**Fig. 1:**
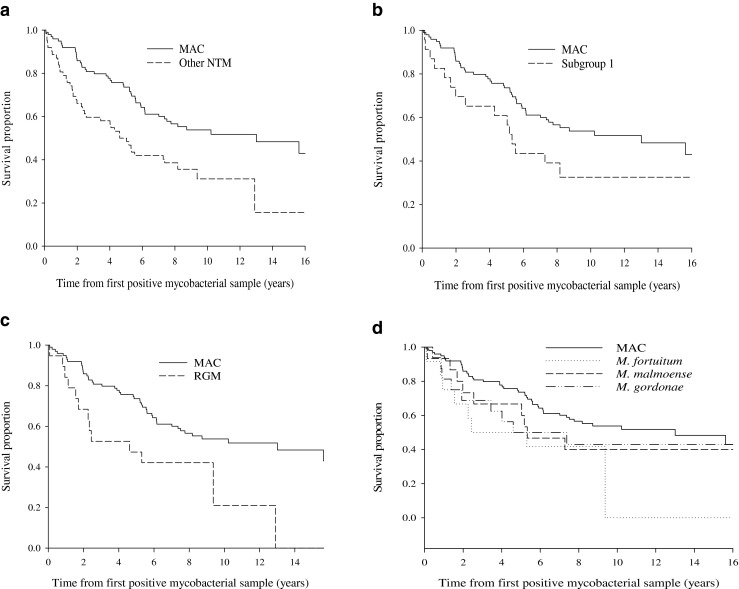
Kaplan–Meier survival curves for patients having at least one pulmonary isolation of various non-tuberculous mycobacteria (NTM). *Panel A*: *Mycobacterium avium* complex (MAC) (*n* = 99) compared to all other NTM species (*n* = 62). The univariate Cox proportional hazards model was applied for the comparison of MAC vs. other NTM; hazard ratio (HR) 0.50, 95 % confidence interval (CI) 0.33–0.77, *p* = 0.002. *Panel B*: MAC (*n* = 99) vs. subgroup 1 of other slowly growing NTM species, including *M. malmoense*, *M. xenopi*, *M. paranifficum*, *M. terrae* and *M. triplex* (*n* = 23). MAC vs. subgroup 1; HR 0.55, 95 % CI 0.31–0.99, *p* = 0.048. *Panel C*: MAC (*n* = 99) vs. a subgroup of rapidly growing mycobacteria (RGM), including *M. fortuitum*, *M. chelonae* and *M. abscessus* (*n* = 19). MAC vs. RGM; HR 0.47, 95 % CI 0.25–0.87, *p* = 0.02. *Panel D*: MAC (*n* = 99) vs. the most common other NTM (*M. fortuitum n* = 12, *M. malmoense n* = 15 or *M. gordonae n* = 16). MAC vs. *M. malmoense*; HR 0.64, 95 % CI 0.31–1.31, *p* = 0.22. MAC vs. *M. gordonae*; HR 0.64, 95 % CI 0.31–1.31, *p* = 0.22. MAC vs. *M. fortuitum*; HR 0.46, 95 % CI 0.22–0.98, *p* = 0.045

The median follow-up time of these patients was 7.0 (range 0.1–24.5) years, without significant differences between the study populations (6.1 years in patients from 1990 to 1998 vs. 7.4 years in patients from 2004 to 2009, *p* = 0.12). The follow-up time was at least 5 years in 63.5 % of all patients (60.0 % in patients from 1990 to 1998 vs. 72.3 % in patients from 2004 to 2009, *p* = 0.14). Patients with pulmonary MAC had a significantly lower risk of death as compared to patients with pulmonary infection of other NTM (HR 0.50, 95 % CI 0.33-0.77, *p* = 0.002, Fig. 1, panel A). The median survival time was 13.0 years (95 % CI 5.9–20.1) for pulmonary MAC patients and 4.6 years (95 % CI 3.4–5.9) for pulmonary other NTM patients (Fig. 1, panel A). Patients with pulmonary MAC also had significantly lower risk of death as compared to patients with pulmonary infection due to other slowly growing NTM (*M. malmonese*, *M. xenopi*, *M. paranifficum*, *M. terrae*, *M. triplex*) (HR 0.55, 95 % CI 0.31–0.99, *p* = 0.048, Fig. 1, panel B. Also, when compared to patients with pulmonary infection caused by RGM, the risk of death was lower with pulmonary MAC patients (HR 0.47, 95 % CI 0.25–0.87, *p* = 0.02, Fig. 1, panel C).

Further, subgroup analysis was done to compare the risk for fatal outcome between pulmonary MAC patients and patients with pulmonary infection due to some defined NTM species. Patients with pulmonary MAC had a significantly lower risk of death as compared to pulmonary *M. fortuitum* (HR 0.46, 95 % CI 0.22–0.98, *p* = 0.045) but not a significantly different risk of death as compared to patients with *M. malmoense* (HR 0.64, 95 % CI 0.31–1.31, *p* = 0.22) or *M. gordonae* (HR 0.64, 95 % CI 0.31–1.31, *p* = 0.22, Fig. 1, panel D).

Among the other NTM group, three analyses were performed using the whole follow-up time and the follow-up time restricted to the first 3 and 5 years. RGM was chosen to be the reference group. There was no significant difference in survival between RGM, *M. gordonae* or *M. malmoense* during the first 3 years (*p* = 0.85). Also, the pairwise comparisons to RGM were non-significant (HR 0.77, 95 % CI 0.26–2.29, *p* = 0.64 and HR 0.77, 0.26–2.28, *p* = 0.63 for *M. gordonae* and *M. malmoense*, respectively). During the first 5 years, the difference was also non-significant (*p* = 0.63), and the pairwise comparisons to RGM were HR 0.99, 95 % CI 0.40–2.47, *p* = 0.99 and HR 0.61, 95 % CI 0.21–1.76, *p* = 0.36 for *M. gordonae* and *M. malmoense*, respectively. During the whole follow-up time, the global test was non-significant (*p* = 0.90) and the pairwise comparisons were also non-significant (HR 0.89, 95 % CI 0.38–2.07, *p* = 0.79 and HR 0.82, 95 % CI 0.36–1.90, *p* = 0.65 for *M. gordonae* and *M. malmoense* as compared RGM, respectively).

**Fig. 2:**
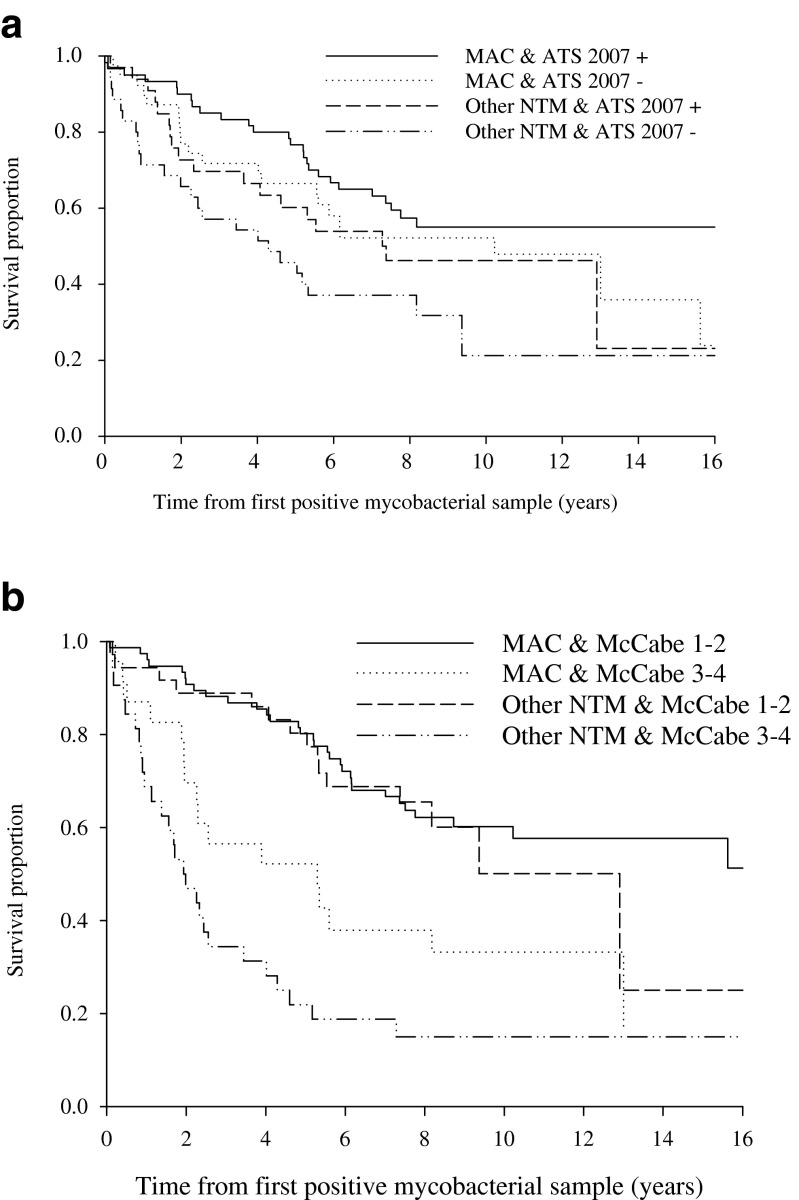
Kaplan–Meier survival curves for all patients with at least one isolation of MAC (*n* = 99) or other NTM species (*n* = 68). *Panel A*: grouped according to American Thoracic Society (ATS) 2007 criteria fulfilment as either positive or negative. The multivariate Cox proportional hazards model yielded a significant effect for MAC (HR 0.57, 95 % CI 0.37–0.88, *p* = 0.01). The effect of ATS 2007 positivity was non-significant (HR 0.66, 95 % CI 0.44–1.01, *p* = 0.06). *Panel B*: grouped according to those with fatal underlying diseases (McCabe 3–4) or without fatal underlying diseases (McCabe 1–2). The multivariate Cox proportional hazards model yielded a significant effect for MAC (HR 0.65, 95 % CI 0.42–1.00, *p* = 0.048) and for McCabe 3–4 (HR 3.33, 95 % CI 2.16–5.12, *p* < 0.001)

When fulfilment of MAC and fulfilment of ATS 2007 criteria were predictors in the same multivariate Cox proportional hazards model, the survival among the MAC group (*n* = 99) was significantly higher than in the other NTM group (*n* = 68) (HR 0.57, 95 % CI 0.37–0.88, *p* = 0.01, Fig. 2, panel A) and, among the ATS 2007-positive patients, the survival tended to be higher than among the ATS 2007-negative patients (HR 0.66, 95 % CI 0.44–1.01, *p* = 0.06, Fig. 2, panel A). The interaction term between these two predictors was non-significant (*p* = 0.89) and was excluded from the multivariate model. Furthermore, when fulfilment of MAC and fatal underlying diseases (McCabe 3–4 vs. 1–2) were predictors in the same multivariate Cox proportional hazards model, the MAC group had significantly better survival than the other NTM group (HR 0.65, 95 % CI 0.42–1.00, *p* = 0.048, Fig. 2, panel B) and patients with McCabe 3–4 had significantly worse survival than patients with McCabe 1–2 (HR 3.33, 95 % CI 2.16–5.12, *p* < 0.001, Fig. 2, panel B). The interaction term between these two predictors was non-significant (*p* = 0.24) and was excluded from the multivariate model. According to univariate analyses, gender, age and smoking were also significant predictors for fatal outcome (HR 0.40, 95 % CI 0.26–0.62, *p* < 0.001 for female gender, HR 1.05, 95 % CI 1.03–1.07, *p* < 0.001 for age in years and HR 1.69, 95 % CI 1.10–2.60, *p* = 0.02 for smoking). Thus, in addition to fulfilment of MAC, the forward stepwise multivariate Cox model (with *p* < 0.05) included gender, age, smoking, McCabe classification and ATS 2007 fulfilment as potential predictors. According to this multivariate stepwise model, the only and equally important predictors, were ultimately or rapidly fatal disease (McCabe 3–4) (HR 3.21, 95 % CI 2.05–5.01, *p* < 0.001), age in years (HR 1.07, 95 % CI 1.04–1.09, *p* < 0.001) and female sex (HR 0.38, 95 % CI 0.24–0.59, *p* < 0.001). Thus, fulfilment of MAC or ATS 2007 criteria did not significantly improve the prediction model when McCabe classification, age and gender were already included.

## Discussion

Knowledge on the prognostic factors in NTM infection is limited. Predictors of mortality among NTM infection are unclear and the pathogenicity of various NTM species may be variable. In this study, we observed that patients with MAC had significantly better prognosis as compared to patients with other NTM infections. Pulmonary MAC patients had significantly lower risk of death as compared to patients with pulmonary infection of other slowly growing NTM and as compared to patients with pulmonary RGM. The main predictors of mortality in our study were both ultimately and rapidly fatal underlying diseases, older age and male gender. The findings are consistent with prognostic factors in recent studies [1, 20]. In another recent study, haemoptysis and consolidation in radiological imaging were also found to be signs of poor prognosis [28].

Somewhat surprisingly, in our study, the patients with other NTM had worse prognosis than MAC patients. In accordance with our results, in a recent study, *M. xenopi* was found to be related to poorer prognosis as compared to MAC [1]. The overall mortality of our MAC patients (median survival of 13.0 years) and other NTM patients (median survival of 4.6 years) corresponded to those observed in recent studies [1, 20]. These results challenge our current view on NTM infections where MAC has been regarded as the main pathogen.

Ultimately and rapidly fatal underlying diseases were the main predictors for mortality and seemed to be the major predisposing factors for infection due to other NTM in our material. Further, MAC patients had significantly less severe underlying diseases and they were more often non-smoking females as compared to other NTM patients. In a subgroup analysis, which included only patients without any fatal underlying diseases, the Kaplan–Meyer survival curves for MAC and other NTM patients were almost superimposable during the 8 years of follow-up (Fig. 2, panel B). Yet, MAC patients who had a fatal underlying disease seemed to have better prognosis than those with isolation of other NTM (Fig. 2, panel B). MAC patients had better outcome than the other NTM patients, even when severe underlying diseases were taken into account in the Cox proportional hazards model analysis. However, it has to be pointed out that 77 % of MAC and 53 % of other NTM patients did not have any fatal underlying diseases.

We could not highlight any single underlying disease behind NTM infections, because we did not test single underlying diseases statistically. However, 74 % of patients in both groups had a previous pulmonary disease. Equally, smoking seemed to be common among our patients. Two-thirds of the other NTM group and less than half of the MAC group were smokers. Smoking was related, for the most part, to male gender, COPD and systemic or inhaled corticosteroid treatment. In a recent study, COPD and corticosteroid treatment was found to be strong risk factors for NTM disease [29]. However, which comes first, the hen or the egg, has evidently not been settled and, at least in some patients, it might be asked whether it is NTM disease or COPD that precedes the other. Namely, NTM infection was suggested to be one of the risk factors for COPD [30]. Heavy smoking history has been previously related to *M. malmoense* and *M. xenopi* infection, but the two other common isolations in our material, *M. chelonae* and *M. fortuitum*, have not been related to smoking to the same extent [8, 17, 23, 31]. Plausibly, smoking is a contributing factor in the prognosis of NTM infection. This could also partly explain the difference in outcome between MAC and other NTM, as MAC patients were more often female and over half of them were non-smokers, as also described in other studies [22, 32, 33]. The result of ATS criteria fulfilment as a predictor of mortality was controversial when compared to their original idea. Although ATS criteria have been used to select patients for antimycobacterial treatment, ATS criteria fulfilment was not a marker for poor but rather improved prognosis in both MAC and other NTM groups (Fig. 2, panel A). This observation is in line with our previous finding with partly the same patient material and also with a larger registry-based material [1, 10]. Unfortunately, one limitation of this retrospective and descriptive study was that we cannot discern what contribution the NTM infection had in the fatal outcome of the patients and which deaths were due to other causes. Plausibly, severe underlying diseases explain the mortality rate in ATS-negative patients. The lack of severe underlying diseases may be one explaining factor behind the better prognosis of MAC (Fig. 2, panel B).

The short time interval from symptom onset in our study suggests, together with the poor prognosis, that NTM infections are more rapid than generally emphasised. Overall, in 43 % of the patients, symptoms had lasted for less than a year and in 75 % less than 2 years before the first positive NTM culture (Table 3). Of the respiratory symptoms in this study, dyspnoea was complained by half of all patients already when NTM was isolated, which is consistent with other studies [34]. The short time frame from symptom onset is consistent with a recent nationwide cohort study, which suggested that respiratory failure may appear in the first 6 months post-diagnosis and the risk is greater among NTM patients with COPD or MAC [16]. Moreover, pulmonary infections due to *M. xenopi* or *M. malmoense* and infections due to *M. chelonae* or *M. abscessus* among organ transplant patients have been reported to progress to death within a few years [8, 23, 29, 35, 36]. Interestingly, other NTM infections in our study had a short symptom duration more often than MAC. Systemic symptoms such as fever, fatigue and lower BMI in our MAC patients would all also comply with the longer disease duration in MAC as compared to other NTM. This would fit into prolonged disease history in MAC because low BMI in NTM disease has been associated to abnormal control of leptin and adipokines, suggesting a prolonged disease history [30, 32].

*M. malmoense* was the third most common NTM isolation, as could be expected and as reported in other studies from Nordic countries [8, 9, 37]. However, RGM (*M. fortuitum*, *M. chelonae*, *M. abscessus*) together formed one-third of all the other NTM isolations. *M. gordonae* was common in cancer patients. It is often regarded a contamination and its role in clinical infections has been questioned, although it has been reported in patients with cancer or immunosuppression [17]. Although our material was small for detailed analyses between NTM strains, we did not observe any difference in prognosis between patients with *M. malmoense*, *M. gordonae* and RGM isolations. Although the precise role of NTM infection for the poor prognosis is still open, it would warrant studies on the effect of medical treatment on prognosis. Namely, in our previous analysis, we could look for the effect of treatment of 3 months with at least two effective drugs and found it to have no effect on prognosis [10]. Accordingly, in a recent analysis on only MAC disease, antimycobacterial therapy was not found to affect prognosis [20]. Clearly, studies on the effect of adequate treatment on the outcome on NTM infection in immunocompetent patients would be needed and the present study cannot give any answer on this. The effect of treatment of severe underlying diseases in the outcome of NTM patients has not been studied either. However, in recent studies, COPD, bronchiectasis and interstitial pulmonary diseases were related to high mortality in patients with NTM infection [15, 38].

Our data show that, together with other recent studies, NTM isolation was a sign of poor prognosis. Pulmonary MAC patients had better prognosis than patients with pulmonary infection due to other NTM. In one-third of MAC patients and a half of other NTM patients, NTM isolation was made within a year of symptom onset, suggesting a fairly rapid disease course. Underlying diseases were the main explanation for poor prognosis. Patients who fulfilled the ATS criteria had better prognosis than ATS-negative patients. The results indicate that the current understanding on the importance of an NTM isolation is insufficient.
